# The antigen-binding fragment of human gamma immunoglobulin prevents amyloid β-peptide folding into β-sheet to form oligomers

**DOI:** 10.18632/oncotarget.17074

**Published:** 2017-04-13

**Authors:** Victòria Valls-Comamala, Biuse Guivernau, Jaume Bonet, Marta Puig, Alex Perálvarez-Marín, Ernest Palomer, Xavier Fernàndez-Busquets, Xavier Altafaj, Marta Tajes, Albert Puig-Pijoan, Rubén Vicente, Baldomero Oliva, Francisco J. Muñoz

**Affiliations:** ^1^ Laboratory of Molecular Physiology, Faculty of Health and Life Sciences, Universitat Pompeu Fabra, Barcelona, Spain; ^2^ Laboratory of Structural Bioinformatics (GRIB), Faculty of Health and Life Sciences, Universitat Pompeu Fabra, Barcelona, Spain; ^3^ Unitat de Biofísica, Departament de Bioquímica i de Biologia Molecular, Facultat de Medicina, Universitat Autònoma de Barcelona, Barcelona, Spain; ^4^ Institute for Bioengineering of Catalonia (IBEC), Barcelona, Spain; ^5^ ISGlobal, Barcelona Centre for International Health Research, Hospital Clínic-Universitat de Barcelona, Barcelona, Spain; ^6^ Bellvitge Biomedical Research Institute (IDIBELL) - Unit of Neuropharmacology and Pain, University of Barcelona, Barcelona, Spain; ^7^ Heart Diseases Biomedical Research Group, IMIM-Hospital del Mar Medical Research Institute, Barcelona, Spain; ^8^ Servei de Neurologia, Hospital del Mar-IMIM-Parc de Salut Mar, Barcelona, Spain

**Keywords:** Alzheimer's disease, amyloid, immunoglobulin, Fab, oligomers

## Abstract

The amyloid beta-peptide (Aβ) plays a leading role in Alzheimer's disease (AD) physiopathology. Even though monomeric forms of Aβ are harmless to cells, Aβ can aggregate into β-sheet oligomers and fibrils, which are both neurotoxic. Therefore, one of the main therapeutic approaches to cure or delay AD onset and progression is targeting Aβ aggregation. In the present study, we show that a pool of human gamma immunoglobulins (IgG) protected cortical neurons from the challenge with Aβ oligomers, as assayed by MTT reduction, caspase-3 activation and cytoskeleton integrity. In addition, we report the inhibitory effect of IgG on Aβ aggregation, as shown by Thioflavin T assay, size exclusion chromatography and atomic force microscopy. Similar results were obtained with Palivizumab, a human anti-sincitial virus antibody. In order to dissect the important domains, we cleaved the pool of human IgG with papain to obtain Fab and Fc fragments. Using these cleaved fragments, we functionally identified Fab as the immunoglobulin fragment inhibiting Aβ aggregation, a result that was further confirmed by an *in silico* structural model. Interestingly, bioinformatic tools show a highly conserved structure able to bind amyloid in the Fab region. Overall, our data strongly support the inhibitory effect of human IgG on Aβ aggregation and its neuroprotective role.

## INTRODUCTION

Alzheimer's disease (AD) is a neurodegenerative process leading to irreversible dementia. The main hallmark of AD is the extracellular accumulation of amyloid β-peptide (Aβ) to form oligomers and fibrils, which are synaptotoxic [1–3] and induce neuronal death [4–6].

Aβ is produced by all the cells of the organism as a result of amyloid precursor protein (APP) proteolytic processing, when it is sequentially cleaved by beta- and gamma-secretase complex [7–10]. Under physiological conditions, Aβ is produced within the brain at a low rate. Aβ length varies depending on its cleaving sites: Aβ_1-40_ is the most abundant, followed by Aβ_1-42_. Additionally, there are larger (1-43) and N-terminal truncated (X-40 and X-42) Aβ species [11]. Once Aβ is released into the brain parenchyma it can be degraded, mostly by neprilysin [12] and insulin-degrading enzyme (IDE)[13]. Nevertheless, there are other proteolytic enzymes contributing to its degradation, such as plasmin [14], the angiotensin converting enzyme (ACE) [15] and endothelin converting enzyme (ECE) [16]. Monomeric Aβ that is not degraded is drained into the blood through the Low-Density Lipoprotein Receptor-Related Protein (LRP), which is in the basal membrane of the endothelium and releases Aβ to plasma by transcytosis [17]. The beta-secretase (BACE1) activity increases with age [18], which causes an increase in Aβ production. Particularly, Aβ_1-42_ levels are increased in the elder, probably due to the nitrotyrosination of gamma-secretase complex [10]. Aβ_1-42_ misfolding into β-sheet favours its aggregation and the formation of oligomers [6], preventing its drainage through the blood brain barrier (BBB). This aggregation also produces cerebral amyloid angiopathy, due to the accumulation of Aβ in brain vessels [19, 20].

Currently, there is no effective treatment for AD. However, different approaches to reduce Aβ deposition are being studied, including the modulation, clearance or inhibition of Aβ aggregation [21]. There are strong evidences showing that the reduction of Aβ could be helpful for treating AD [22, 23]. In this context, there are reports on immunotherapy, which relies on evidences showing that Aβ antibodies not only inhibit fibril formation, but also disassemble pre-formed aggregates [24, 25]. Gamma immunoglobulins (IgG) can cross the BBB to act in the central nervous system [26]. Different groups have studied the mechanism by which antibodies targeting Aβ N-terminal region inhibit its aggregation [24, 27, 28]. In addition, it has been shown that peripheral administration of anti-Aβ antibodies could reduce brain plaques in an AD mouse model [26]. Several clinical trials using humanized monoclonal antibodies against Aβ, showed promising results regarding safety and tolerance [21, 29–33]. Although none of them has overcome phase III for mild to moderate AD until now [30, 31], some trials are still ongoing because of their potential [32, 34, 35]. The administration of an intravenous human serum IgG preparation was reported to be safe, and the treatment was confirmed to be present in the cerebrospinal fluid (CSF). Even though the treatment was effective among APOE-e4 carriers, it was not able to overcome phase III [36, 37]. Recently, the humanized IgG aducanumab has obtained very promising results, demonstrating a decrease in brain Aβ that correlates with slow clinical decline [32].

Here, we report the role of the human pool of IgG in the inhibition of Aβ aggregation and neurotoxicity, independently of IgG antigen-specificity. Particularly, we show experimentally for the first time the importance of the antigen-binding fragment (Fab) in the inhibition of Aβ aggregation.

## RESULTS

### IgG prevents amyloid toxicity in cortical neurons

**Figure 1:**
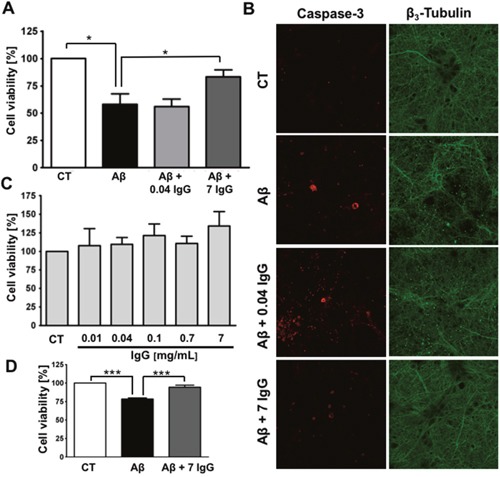
IgG protects cortical neurons against amyloid toxicity **(A)** Cell viability was assayed by MTT reduction in cortical neurons treated with 10 μM Aβ_1-42_ or 10 μM Aβ_1-42_ + IgG at either 0.04 or 7 mg/mL for 24 h. Data are the mean ± SEM of 6 independent experiments. *p < 0.05 by one-way ANOVA using Newman-Keuls post-test. **(B)** Immunofluorescence study of cortical neurons treated with 10 μM Aβ_1-42_, 10 μM Aβ_1-42_ + 0.04 mg/mL of IgG or 10 μM Aβ_1-42_ + 7 mg/mL of IgG for 24 h. Staining for active caspase-3 is shown in red and for β_3_-tubulin in green. β_3_-tubulin is an overlap of images of the same area **(C)** Cell viability was assayed in cortical neurons by MTT reduction treated with increasing concentrations of IgG for 24 h. Data are the mean ± SEM of 4 independent experiments. **(D)** Cell viability was assayed by MTT reduction in endothelial cells treated with 0.1 μM Aβ_1-40_ or 0.1 μM Aβ_1-40_ + IgG 7 mg/mL for 24 h. Data are the mean ± SEM of 5 independent experiments. *** p < 0.001 by one-way ANOVA using Newman-Keuls post-test.

Ultimately, the deposition of Aβ in AD brain leads to neuronal death. Therefore, we have tested IgG effect on Aβ-mediated neurotoxicity. Aβ oligomers, resulting from the aggregation of Aβ_1-42_, were prepared in the presence or absence of IgG, either at plasmatic physiological concentration (7 mg/mL) or at 0.04 mg/mL because it has been reported as the non-pathological IgG concentration in the central nervous system (CNS) [38]. We used Aβ_1-42_ because it is the most aggregation-prone species and, in consequence, the most neurotoxic amyloid form [39]. The different treatments were administered to cortical primary neurons to further evaluate cell viability. Aβ_1-42_ oligomers-mediated neurotoxicity was significantly reduced in the presence of 7 mg/mL of IgG, but not with IgG at 0.04 mg/mL (Figure 1A). To confirm this observation, we analyzed apoptosis performing an immunofluorescence staining against active caspase 3 and β_3_-tubulin (Figure 1B). We observed higher levels of active caspase 3 and an altered neuronal morphology, characterized by less neurites in neurons treated with Aβ_1-42_ oligomers prepared in the absence of IgG. Conversely, Aβ_1-42_ oligomers prepared in the presence of IgG at 7 mg/mL prevented the increase in caspase 3 and preserved the morphology. No protective effect was observed when IgG at 0.04 mg/mL was co-incubated with Aβ_1-42_. Controls with different concentrations of IgG showed no effects on the viability of cortical neurons (Figure 1C).

Considering that cerebral amyloid angiopathy is a concomitant pathology in most of AD cases [40, 41], we assayed the effect of IgG on endothelial protection against amyloid challenge (Figure 1D). Brain vascular amyloid deposits are mainly composed of Aβ_1-40_ [42], in consequence, we used this species to perform the experiment. The treatment was performed using a final concentration of 0.1 μM because it produces a significant decrease in cell viability. As expected, simultaneous incubation of Aβ_1-40_ with 7 mg/mL of IgG protects endothelial cells against oligomers cytotoxicity.

### IgG inhibits Aβ aggregation

**Figure 2:**
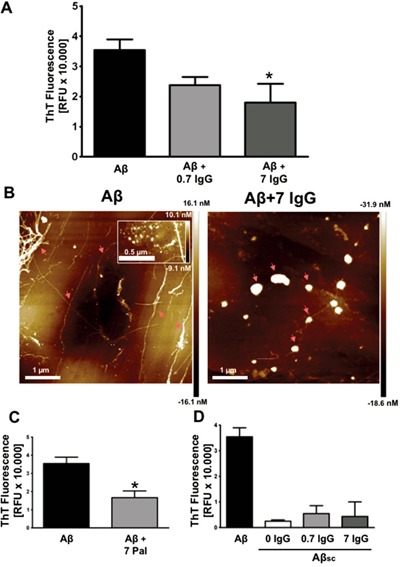
IgG inhibits amyloid aggregation **(A)** Aggregation assay of 27 μM Aβ_1-42_ with or without IgG at 0.7 and 7 mg/mL during 96 h. ThT fluorescence was measured at 96 h. Data are the mean ± SEM of 3 independent experiments. * p < 0.05 compared to control by one-way ANOVA using Newman-Keuls post-test. **(B)** AFM images of 88,6 μM Aβ_1-42_ (left) and 88,6 μM Aβ_1-42_ treated with 7 mg/mL of IgG (right) for 96 h. The inset in the left image shows a cluster of oligomers. Arrows indicate mature fibers (left) and amorphous globular structures (right) **(C)** Aggregation assay of 27 μM Aβ_1-42_ with or without Palivizumab at 7 mg/mL during 96 h. ThT fluorescence was measured at 96 h. Data are the mean ± SEM of 3 independent experiments. * p < 0.05 by t-student test. **(D)** Aggregation assay of 27 μM Aβ_1-42_, 27 μM Aβ_1-42_ scramble (Aβs_c_) and 27 μM Aβs_c_ co-incubated with IgG at 0.7 mg/mL and 7 mg/mL. ThT fluorescence was measured at 96 h. Data are the mean ± SEM of 3 independent experiments.

After demonstrating that IgG prevents Aβ-mediated neurotoxicity, we were interested in elucidating the mechanism by which IgG mediates its protective effect. Therefore, Aβ_1-42_ was set at aggregation conditions in the presence or absence of IgG (Figure 2A). We used the plasmatic physiological concentration of IgG (7 mg/mL) and a subphysiological concentration (0.7 mg/mL). Thioflavin T (ThT) fluorescence was analyzed after 96 h of aggregation assay to allow fibril formation. We observed a significant reduction of Aβ_1-42_ fibril formation in the presence of 7 mg/mL of IgG (p<0.05). To confirm these results, Aβ1-42 was visualized on freshly cleaved highly oriented pyrolytic graphite (HOPG) with atomic force microscopy (AFM; Figure 2B). In the untreated Aβ1-42 controls after 96 h of aggregation mature fibers could be clearly identified, as well as clusters of oligomers (inset in Figure 2B), whereas after co-incubation with 7 mg/mL of IgG, there were mostly amorphous globular structures concomitant with a significant reduction of mature fibrils. Overall, these results are in agreement with the data obtained in ThT assays.

To study the importance of antibody's specificity we used Palivizumab (Pal), a humanized IgG against respiratory syncytial virus [43] (Figure 2C). Aβ_1-42_ was aggregated in the presence of Pal (7 mg/mL). The aggregation process was analyzed by ThT fluorescence after 96 h. Our results show a significant decrease in Aβ_1-42_ fibril formation similar to the observed with the pool of human IgG (Figure 2A). Thus, the inhibition of amyloid aggregation *in vitro* by IgG seems to be independent of IgG specificity.

In order to rule out an unspecific effect of IgG, we measured ThT fluorescence in an aggregation assay of scramble Aβ_1-42_ (Aβ_sc_; which is not able to aggregate) and Aβ_sc_ co-incubated with IgG at 7 mg/mL and 0.7 mg/mL (Figure 2D). Data showed the specificity of ThT fluorescence on fiber formation and the absence of inherent fluorescence of IgG.

**Figure 3:**
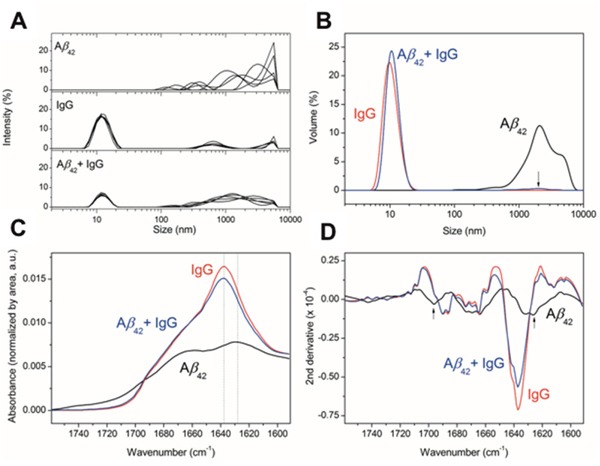
Effect of IgG on Aβ oligomerization **(A)** DLS. Size distribution measured by DLS as a function of signal intensity of particles for 5 DLS measurements of 10 scans each. **(B)** Size distribution as a function of volume of the measured particles for the averaged measurements. The arrow indicates the presence of a low percentage population at the specified size for the mixture of Aβ plus IgG. **(C)** ATR-FTIR spectroscopy of the amide I region for the indicated samples in a thin hydrated film. All spectra are normalized by the area under the curve, representative of the protein amount measured in each thin hydrated film. The dotted lines indicate the wavenumber position of the highest intensity peaks for the samples analyzed. **(D)** Second derivative of the ATR-FTIR to highlight different secondary structures due to mathematical band narrowing [59]. The arrows indicate the position of the bands characteristic for β-sheet amyloid aggregation.

Finally, the presence of different amyloid aggregates populations was analyzed by dynamic light scattering (DLS, Figure 3A and 3B) and the corresponding secondary structure was characterized using attenuated total reflection Fourier transform infrared spectroscopy (ATR-FTIR, Figure 3C and 3D). Aβ_1-42_ oligomers measurement by DLS showed a detectable population of high molecular weight (particles > 1 μm) and even larger aggregates, which could not be detected and that were over the device detection limit (sharp decay at sizes > 5 μm, top panel Figure 3A). For IgG, there is a major population (ca. 17%) centered at around 13 nm, corresponding to the size of antibodies, and two less populated (<5%) fractions 500 nm and 5 μm (middle panel Figure 3A). Aβ_1-42_ oligomers incubated with 7 mg/ml of IgG for 24h show two populations, one centered at 13 nm corresponding to IgG and a second one widely centered at 1 μm (lower panel Figure 3A). In Figure 3B, the size distribution as a function of particle volume shows that the sample containing Aβ_1-42_ oligomers has a dominant population at > 1 μm, whereas in the Aβ_1-42_ oligomers incubated with 7 mg/ml of IgG sample two populations appear, the IgG at 13 nm and a minor population at 2 μm. The concentration of IgG (7 mg/ml) compared to Aβ_1-42_ oligomers (0.4 mg/ml) may mask the observation of aggregation in DLS, thus we performed ATR-FTIR in thin hydrated films, normalizing the signal for the protein concentration present in the film (Figure 3C). The Aβ_1-42_ oligomers spectrum shows a distinctive amyloid β-sheet band at 1628 cm^-1^, which is absent in the IgG containing samples. To confirm that amyloid β-sheet bands were absent in the IgG containing samples, band narrowing using the spectra second derivative is shown in Figure 3D. None of the β-sheet bands is present in sample Aβ_1-42_ oligomers co-incubated with 7 mg/ml of IgG. The results obtained by DLS and ATR-FTIR further support that IgG prevents Aβ_1-42_ aggregation *in vitro*. Overall, these experiments suggest a protective effect of IgG against Aβ_1-42_ aggregation and toxicity.

### Fab fragment is involved in the inhibitory effects in Aβ aggregation

**Figure 4:**
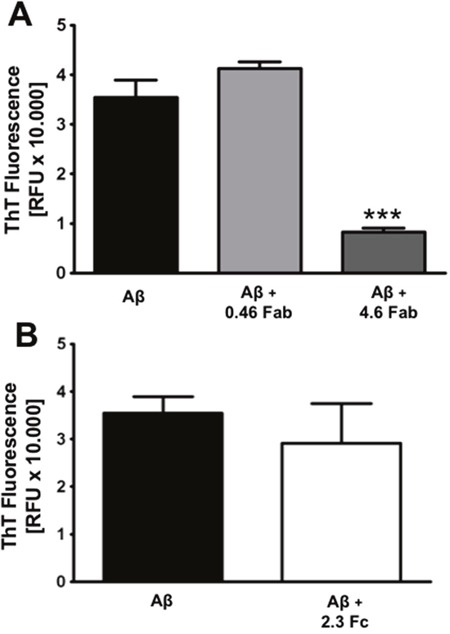
Fab is the region that inhibits amyloid aggregation Aggregation assay of 27 μM Aβ_1-42_ co-incubated with Fab fragment at 0.46 and 4.6 mg/mL **(A)** or Fc fragment at 2.3 mg/mL **(B)** during 96 h. ThT fluorescence was measured at 96 h. Mean ± SEM of 3 independent experiments *** p < 0.001 by one-way ANOVA using Newman-Keuls post-test.

The IgG is formed by the Fab, which contains the antigen-binding site and it is responsible for IgG specificity and the crystallizable fragment (Fc). Our next step was to study whether IgG inhibitory effect in Aβ_1-42_ aggregation and neurotoxicity was a property of the whole IgG or it relies on one of these fragments. Therefore, Aβ_1-42_ was co-incubated, separately, with the Fab and with the Fc for 96 h and ThT fluorescence was measured. Fab and Fc concentrations were calculated based on IgG stoichiometry. We observed that Fab inhibits Aβ_1-42_ aggregation in a concentration-depending manner (Figure 4A), suggesting that it is the region that binds to amyloid. On the other hand, Fc did not show any effect in Aβ_1-42_ aggregation (Figure 4B).

**Figure 5:**
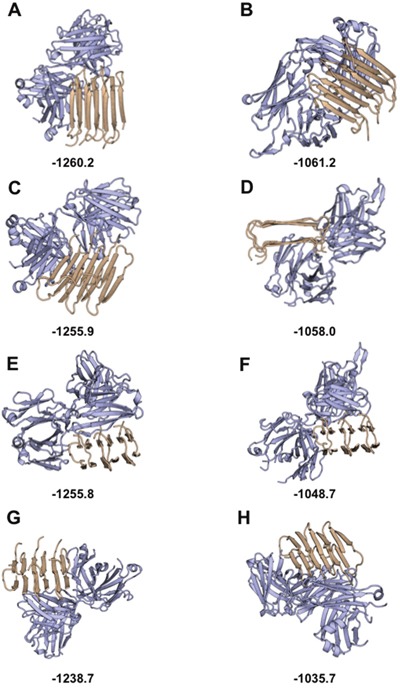
*In silico* study of Aβ_1-42_ binding the IgG Fab region: Top scored decoys for antibody-Aβ_1-42_ docking *In silico* structural modeling of the interaction between amyloid (light brown) and the functional region of the Fab domain (light blue). Top scored decoys for the interaction with the structure of the Fab region of myeloma IgG are shown in **(A, C, E**, and **G,)** with best to worst decoys, respectively. Top scored decoys for the interaction with the structure of the Fab region of HIV IgG are shown in **(B, D, F**, and **H,)** with best to worst decoys, respectively. Scores obtained with Rosetta are shown at the bottom of each decoy (low score Rosetta-energies indicate the most stable decoys).

To explore the mechanism for non-specific binding of Fab-Aβ_1-42_, a rigid-body docking analysis was performed between a selected group of non-redundant human Fabs with known structure and the structure of Aβ_1-42_ [44]. The top scored decoys show the Aβ_1-42_ peptide in complex with the human myeloma IgG [45] (Figure 5A) and the human HIV-neutralizing antibody 2F5 [46] (Figure 5B). In both cases, Aβ_1-42_ bound through a non-specific β–β ladder strands-pairing, like the intra-strand interactions of Aβ_1-42_ peptides in fibrillation. This configuration seems favorable, especially in the case of human myeloma IgG, because the stacked β–β pairing with IgG has a Cα-Cα distance of 10.8±0.4Å like the Aβ_1-42_ stacking distance in fibrillation but higher (with more distance between the stacked β–strands) than other exposed β–strands of IgG (8.5±1Å). Combined with experimental data, this model suggests that IgG domains can affect fibrillation by binding Aβ_1-42_ peptides like a new monomer of the fiber, blocking its progression. Considering that fibrillation of Aβ_1-42_ progresses from both strand sides, this mechanism can also explain the concentration-dependence of the process, requiring two IgG to block each side of fiber progression (see Figure 5 and Video: https://drive.google.com/drive/folders/0B_99pS9URra_U2dZS2dxRUJFWnM?usp=sharing).

## DISCUSSION

Different strategies have been used to control Aβ levels in the brain, although none of them has been successful. Nowadays, there are a few drugs approved for AD treatment. Among them, most of the approved drugs for AD are cholinesterase inhibitors (tacrine, donezepil, galantamine, rivastigmine) and also, a NMDA receptor antagonists (memantine) [47]. However, currently there are not drugs capable of modulating the mechanisms of Aβ oligomerization for therapeutic purposes.

The present work shows that the pool of human IgG protects cortical neurons and endothelial cells against Aß cytotoxicity, and inhibits Aβ aggregation at 7 mg/mL. The inhibitory effect on Aß aggregation had already been described for some anti-Aβ antibodies [24]. The authors attribute this effect to its specific binding to Aβ, however it was not shown in which IgG fragment it resides. Here, we identify for the first time Fab as the fragment responsible for inhibition Aβ aggregation, and that this effect is independent of IgG specificity. Over last years, immunotherapy has been considered a promising approach to prevent amyloid plaque formation, and therefore to ameliorate cognitive decline in AD patients. Different clinical trials have been performed, but none of them have been able to overcome phase III [30, 31]. Nonetheless, there are still some anti-Aβ antibodies ongoing phase III trials due to their potential therapeutic effect [32, 34, 35]. The vast majority of treatments that have been tested following this strategy are antibodies targeting Aβ, our results using a pool of human IgG and Palivizumab (Pal) suggest that the specificity of the antibody is not especially relevant for inhibiting *in vitro* amyloid aggregation. However, our results were obtained by IgG or Pal co-incubation with soluble Aβ, and therefore recognizing its native non-folded sequence. Thus, we cannot discard that human anti-Aβ antibodies recognizing oligomers or fibrils folded domains could produce beneficial results in AD treatment.

Clinical trials have shown that immunotherapy reduce Aβ amyloid deposits in the brain, besides showing safety and tolerance [21, 29–33]. However, they also present several significant side effects that should be considered. These side effects, such as cerebral amyloid angiopathy (CAA)-associated cerebral hemorrhage [48] or vascular edema [49], are primarily related to inflammation [50, 51]. Taking into consideration that Fc is the fragment responsible for triggering the immune response, administration of IgG could be a method to overcome inflammatory problems. Moreover, reducing IgG size could be a manner to potentiate their pass through BBB. In addition, intravenous IgG therapy has also been proposed as a treatment for AD. It has been shown to reduce Aβ levels in the CSF, increase its levels in serum and improve cognitive function, but it has not been able to conclude any clinical trial [36, 37, 52]. Importantly, adverse reactions to this treatment are not common and they are usually minor, including mild to moderate headache, fever, chills and myalgia [53]. Nevertheless, a quite promising study on a human anti-Aβ antibody able to recognize oligomers of Aβ has been recently published [32].

On the other hand, amyloid is produced by all the cells of the body [54], but paradoxically, Aß aggregation is just observed inside the brain. These evidences suggest that amyloid aggregation would be physiologically inhibited in systemic tissues and in brain, until the amount of amyloid exceeds a concentration threshold. Is in this scenario that IgG would be acting as a physiological inhibitor of amyloid aggregation, and therefore ameliorating AD condition.

In conclusion, the data presented here contribute to the understanding of the role of IgG regarding Aβ aggregation. Further studies on Fab fragment may lead to the development of new approaches able to prevent Aβ oligomerization without leading to inflammation or other side-effect, hence being a promising treatment for AD.

## MATERIALS AND METHODS

### Mouse cortical primary cultures

Mouse cortical neurons were isolated from 18-day-old CD1 embryos. The procedure was approved by the Ethics Committee of the Institut Municipal d’Investigacions Mèdiques-Universitat Pompeu Fabra (EC-IMIM-UPF). Cortex was aseptically dissected in ice-cold HBSS (Life Technologies) supplemented with 4.5 g/L glucose (Sigma-Aldrich) and trypsinized for 17 min at 37°C. After 3 washes in HBSS+glucose and mechanical dissociation, cells were seeded on DMEM (Life Technologies) plus 10% horse serum (Life Technologies) onto 1% poly-D-Lysine (Sigma-Aldrich, USA) coated plates. After 2 h, medium was removed and Neurobasal medium (Life Technologies) was added containing 2% B27 supplement (Gibco BRL), 1% GlutaMAX (Life Technologies) and 1% Penicillin/Streptomycin. On day 3 of culture (DIV), cells were treated with 2 μM 1-β-D-arabinofuranosylcytosine (AraC; Sigma) for 24 h to eliminate proliferating non-neuronal cells. Primary cortical neurons were used at 10 DIV.

### Porcine aortic endothelial cultures

Endothelial cells were isolated from porcine aortas provided by FRISELVA (Girona, Spain). Endothelial cells were obtained following Epithelial Cell Culture Protocols of Methods in Molecular Biology. Briefly, porcine aortas were cleaned in washing solution (HBSS with 0.002 mM HEPES and antibiotics) and incubated in collagenase solution (50 mg/mL collagenase type II, 0.1 g/L MgCl2·6H2O, 7 g/L NaCl, 0.1 g/L Na2HPO4·7H2O, 0.18 g/L NaH2PO4·H2O, 0.54 g/L D-Glucose and 0.2 % FBS) for 15 minutes. Aortas were scratched into Endothelial Medium (DMEM F12K, 0.1 mg/mL heparin, 10% fetal bovine serum (FBS) and 2 mM of L-Glutamine plus antibiotics). Cells were collected by centrifugation and seeded in T-75 flasks. Cells were grown in Endothelial Medium and were used up to passage number 8.

### Aβ_1-42_ and Aβ_1-40_ preparation

Lyophilized Aβ_1-40_ or Aβ_1-42_ (Anaspec) were solubilized as previously described [55]. Briefly, 1 mg of Aβ was dissolved in 250 μL of MilliQ water and pH was adjusted to ≥ 10.5 using 1 M NaOH solution to avoid the isoelectric point of Aβ. Then, 250 μL of 20 mM Phosphate Buffer (pH 7.4) were added and the preparation was placed for 1 min in a bath-type sonicator (Bioruptor, Diagenode). Aβ preparation was immediately used for oligomer preparation or stored in 25 μL aliquots at -20°C until used for ThT experiments. For oligomer preparation, Aβ was dissolved to 0.4 mg/mL in DMEM/F12 without phenol red with or without IgG (Grifols) or 6E10 (Covance).

### IgG preparation

The pool of human IgG was provided by Grifols (Flebogamma® 5% DIF; 100 mL).

### Cell viability assay by MTT reduction

Cortical primary neurons and endothelial cells were seeded in a 96-well plate at a density of 1×10^4^ cells/well. The treatment (100 μL/well) was added to Neurobasal without phenol red supplemented with 1% GlutaMAX (Life Technologies) for cortical neurons and Endothelial Medium without FBS for endothelial cells. Cells were treated for 24 h at 37°C. Cell viability was tested by 3-(4,5-dimethylthiazol-2-yl)-2,5-diphenyltetrazolium bromide (MTT) reduction. Briefly, 11 μL of MTT stock solution (Sigma Aldrich; 5 mg/mL) were added. After 2 h the media was replaced with 100 μL of DMSO. MTT absorbance was determined in an Infinite 200 multiplate reader (Tecan) at A540 nm and corrected by A650 nm. Untreated cells were taken as 100%.

### Immunofluorescence

Cortical primary neurons (1×10^5^ cells/well) were fixed with 4% paraformaldehyde and permeabilized with 0.1% Triton X-100 for 10 min at RT. Samples were blocked with 5% Fetal Bovine Serum, 1% albumine from bovine serum and 0.02% sodium azide overnight at 4°C. Subsequently, coverslips were incubated for 2 h at RT in a hydration chamber with the following primary antibodies: anti-β_3_ tubulin (1:200; Cell Signalling) and anti-cleaved Caspase-3 (1:100; Abcam). Then, samples were washed thrice with PBS and incubated with Alexa Fluor 488 goat anti-mouse and Alexa Fluor 555 goat anti-rabbit antibodies (1:1000; Life Technologies) for 1 h at RT. Coverslips were mounted and analyzed using a Leica TCS SP5 confocal microscope (63x objective) and analyzed with Leica confocal software.

### Thioflavin T binding assay

A 1 mM stock ThT (Sigma Aldrich) solution was prepared dissolving the dye in PBS. The solution was filtered using a 0.22 μm syringe filter. ThT solutions were stored at -20°C in the dark. Aβ_42_ peptide (27.5 μM) was incubated with or without IgG (0.7 and 7 mg/mL; Grifols), Palivizumab (7 mg/mL; Synagis), Fab (4.6 mg/mL; Grifols) and Fc (2.3 mg/mL; Grifols) and 10 μM ThT in a Nunc-96-well flat bottom black polystrol microplate (Thermo Scientific) at 37°C. ThT fluorescence was measured at 96 h using excitation and emission wavelengths of 430 and 470 nm, respectively, using a multiplate reader fluorimeter (FLUOstar optima, BMG labtech).

### Atomic force microscopy

A MultiMode atomic force microscope (Veeco Instruments Inc., Santa Barbara, CA) equipped with a 12 mm scanner (E-scanner) was used to analyze Aβ samples aggregated with or without IgG for 96 h. Sample aliquots were frozen and were thawed at RT just before the AFM experiments. The images were taken in liquid with a liquid cell without the O-ring seal. 5-10 mL of sample were deposited on cleaved mica substrates. After an adsorption time of 5 min, 40 mL of double deionised water were added to form a drop suitable for the imaging procedure. The system was left for equilibration for at least 10 min before carrying out the experiments. NP-S (Veeco) probes were used to scan the samples in tapping mode at 0.5 Hz scan rate. Height and amplitude images were recorded simultaneously, although only the latter are presented in this paper.

### DLS measurements

Samples were prepared as indicated above and kept for 24 h at 4°C. DLS measurements were recorded on a Zetasizer instrument (Nano ZS; Malvern Instruments) at 20°C using a small volume (40 μL) quartz cuvette of 1 cm path length. Scattering data were collected as an average of 5 measurements with 10 scans for each measurement. Refractive indices were 1.330 (water) and 1.45 (protein) for polydispersant and material, respectively. Data were processed with the Malvern Zetasizer Software (Malvern Instruments).

### ATR-FTIR spectroscopy

Thin hydrated films of samples placed on top of the ATR diamond crystal were prepared under a N2 stream gentle drying. Spectra were obtained at RT by the average of 250 scans at 2cm-1 resolution in a VARIAN FTS-7000 infrared spectrometer. The second derivative was performed with ten smoothing points.

### Size exclusion chromatography

Size exclusion chromatography was performed in an AKTA purifier FPLC system. All chromatograms were run at 4°C using a flow of 0.4 mL/min in 10 mM Tris·HCl buffer pH 7.4. An initial run in a Superdex 75 10/30 column was performed to separate HMW and LMW aggregates. HMW aggregates were injected in a Superose 6 10/30 column to analyze the size exclusion profile of the non-resolved species.

### Fab and Fc fragments purification

To obtain Fab and Fc fragments of IGIV Flebogamma® DIF 5% 100 mL, the commercial Fab preparation kit (Pierce) was used following manufacturer instructions and the molecular distribution analysis method MA_IG-000158A was used. Briefly, 500 μL of IGIV (8 mg/mL) were desalted by Zeba™ Spin Desalting Column following the kit instructions, while 250 μL agarose resin with immobilized papaine, previously packed in a 0.8 mL column, were equilibrated with a digestion buffer (cysteine·HCl 3.5 mg/mL pH 7). Once equilibrated, 500 μL of desalted IGIV were added and maintained in agitation at 900 rpm for 10 h at 37°C. Then, the microcolumn was centrifuged and washed with PBS at 5000 g for 1 min to separate completely the digest and the agarose resin.

To purify the Fab fragment from de digest, it was added to a NAb™ Protein A Spin Column, previously equilibrated, and incubated for 10 min at RT. After the incubation, Fc fragments and undigested IgGs were retained at the column, while Fab purified fragments were collected with flow through. To recover Fc fragments and undigested IgGs, NAb™ Protein A Spin Column was eluted. The eluted was concentrated to 6 mg/mL of protein using Ultracel YM-3 filters 3 kDa NMWL (Millipore). Then, it was injected to an exclusion molecular column TSKgel G3000 SW 60 cm X 7.5 mm. I.D. (Teknokroma) in order to be fractioned with a high-performance liquid chromatography (HPLC) Waters 2695 Separations Module (Alliance) with UV detector diodo-array Waters 996. After 16.5 min, Fc purified fraction was recollected for 1 min. Finally, Fab and Fc purified fragments were concentrated using Amicon Ultra-15 filters 3 kDa NMWL (Millipore).

### Computational analysis of the IgG-amyloid interaction

All human IgGs with less than 70% sequence identity and known structure were retrieved with the Protein Data Bank REST service [56]. From this search, two non-redundant representatives of the IgG Fab region were found: the human myeloma IgG (PDB: 8FAB) [45] and the human HIV-neutralizing antibody 2F5 (PDB:2F5B) [46]. These two structures were tested individually by performing rigid-body docking with the structure of Aβ_1-42_ obtained by solid-state NMR (PDB: 2LNQ) [44].

Around 19000 decoys of the complex IgG-Aβ_1-42_ were obtained for each IgG (2LNQ-8FAB and 2LNQ-2F5B) with PatchDock [57]. We selected the top 100 decoys of PatchDock to calculate the binding ΔG with Rosetta [58]. We selected the top 4 decoys with higher binding affinity for each tested IgG.

The final selected models depict the conformations of Aβ_1-42_ interacting with the human myeloma IgG (Figures 5A, 5C, 5E, 5G) and the human HIV-neutralizing antibody (Figures 5B, 5D, 5F, 5H). The best scored decoys (Figures 5A, 5B) show the interaction between Aβ_1-42_ and the IgG Fab region through the pairing of β-strands, extending the Ig-like fold core β-sheet. The non-specific pairing of β-strands can be seen in other top scored decoys (Figures 5C, 5F, 5G, 5H), although in these decoys only one β-strand of Aβ_1-42_ binds the β-sheet of the IgG fold, reinforced with other side-chain contacts. All decoys show the molecular mechanisms by which the IgG Fab region blocks the progression of the fiber: either playing the role of a new Aβ_1-42_ monomer, or avoiding the attachment of new Aβ_1-42_ monomer due to steric hindrances.

### Statistical analysis

Data are expressed as mean ± SEM of n experiments as indicated in the corresponding figures. Statistical analyses were performed by one-way ANOVA or t-student using GraphPad software. * p < 0.05; *** p < 0.001.

## Abbreviations

Ab: Antibody
Aß: Amyloid-ß peptide
AD: Alzheimer disease
CSF: cerebrospinal fluid
FBS: fetal bovine serum
IgG: gamma-globulin
MTT: 3-(4,5-dimethylthiazol-2-yl)-2,5-diphenyltetrazolium bromide; Palivizumab, Pal
